# Single cell RNA-seq reveals genes vital to in vitro fertilized embryos and parthenotes in pigs

**DOI:** 10.1038/s41598-021-93904-3

**Published:** 2021-07-13

**Authors:** Zhi-Qiang Du, Hao Liang, Xiao-Man Liu, Yun-Hua Liu, Chonglong Wang, Cai-Xia Yang

**Affiliations:** 1 College of Animal Science, Yangtze University, Jingzhou, 434025 Hubei China; 2 College of Animal Science and Technology, Northeast Agricultural University, Harbin, 150030 Heilongjiang China; 3 Key Laboratory of Pig Molecular Quantitative Genetics of Anhui Academy of Agricultural Sciences, Anhui Provincial Key Laboratory of Livestock and Poultry Product Safety Engineering, Institute of Animal Husbandry and Veterinary Medicine, Anhui Academy of Agricultural Sciences, Hefei, 230031 China

**Keywords:** Biotechnology, Developmental biology, Zoology

## Abstract

Successful early embryo development requires the correct reprogramming and configuration of gene networks by the timely and faithful execution of zygotic genome activation (ZGA). However, the regulatory principle of molecular elements and circuits fundamental to embryo development remains largely obscure. Here, we profiled the transcriptomes of single zygotes and blastomeres, obtained from in vitro fertilized (IVF) or parthenogenetically activated (PA) porcine early embryos (1- to 8-cell), focusing on the gene expression dynamics and regulatory networks associated with maternal-to-zygote transition (MZT) (mainly maternal RNA clearance and ZGA). We found that minor and major ZGAs occur at 1-cell and 4-cell stages for both IVF and PA embryos, respectively. Maternal RNAs gradually decay from 1- to 8-cell embryos. Top abundantly expressed genes (CDV3, PCNA, CDR1, YWHAE, DNMT1, IGF2BP3, ARMC1, BTG4, UHRF2 and gametocyte-specific factor 1-like) in both IVF and PA early embryos identified are of vital roles for embryo development. Differentially expressed genes within IVF groups are different from that within PA groups, indicating bi-parental and maternal-only embryos have specific sets of mRNAs distinctly decayed and activated. Pathways enriched from DEGs showed that RNA associated pathways (RNA binding, processing, transport and degradation) could be important. Moreover, mitochondrial RNAs are found to be actively transcribed, showing dynamic expression patterns, and for DNA/H3K4 methylation and transcription factors as well. Taken together, our findings provide an important resource to investigate further the epigenetic and genome regulation of MZT events in early embryos of pigs.

## Introduction

Early embryo development in mammals is one of the most marvelous biological events, and has been studied in great detail in developmental, reproductive and stem cell biology. It debuts from the fertilization of mature oocytes at metaphase of the second meiosis (MII) by sperms, or the parthenogenetical activation by physical and/or chemical stimuli^1^. Maternal factors deposited in oocytes during oogenesis, growth and maturation can reprogram and initiate the transcription of embryo genome^2^. These epigenetic reprogramming events include DNA/histone methylation, chromatin modifications at both local accessibility (histone methylation or acetylation) and spatial configuration (3D chromosomal organization)^3^, which could interact with environmental factors (nutrients, hormones, etc.) to further modify zygotic genome activation (ZGA). The clearance of maternal mRNAs during maternal-to-zygote transition (MZT) in a timely fashion is inevitable to the onset of early embryo development, especially the subsequent ZGA^2,4–6^. When abnormal maternal mRNA clearance happens, ZGA could be impaired, and embryo development will fail^6^.

The mammalian ZGA event is species-specific, and occurs in two waves (minor and major)^7^. In mice, the minor and major ZGA are characterized to be at 1-cell and 2-cell stages, respectively^8^. However, in pigs, the major ZGA is considered to be at the 4-cell to 8-cell stages^9–12^. A recent study confirmed the timing of major ZGA, employing the single-cell RNA sequencing (scRNA-seq) method on pig early in vivo fertilized embryos, to dissect the molecular features of pluripotency, dynamics of X-chromosome dosage compensation and the first lineage specification^13^. Using only trace amount of materials, the powerful scRNA-seq can profile the transcriptome dynamics^14^, and has been used to reveal the transcriptome maps of early embryos in humans^15^, mice^16^ and monkeys^17^. However, in pigs, majority of RNA-seq studies used pooled whole early embryos derived from in vitro and in vivo fertilization, and somatic cell nuclear transfer^18,19^. Only few scRNA-seq studies have been performed^13^, including our works on screening pig oocytes with better quality for maturation^20^, and aberrant signaling pathways underlying oocyte maturation when perturbed by chemical treatment^21^. Even though we already know that embryos developed by in vitro fertilization or parthenogenetic activation possess poor quality and developmental competence^1^, the exact molecular mechanisms are still poorly understood.

Genome-wide transcript profiling during MZT in pigs could contribute to understanding stage-specific transcriptome, the clearance of stored maternal transcripts, and newly synthesized transcripts in ZGA, thereby identifying key genes and associated signaling pathways. In the present study, using scRNA-seq, we studied comparatively the transcriptomes of porcine oocytes and single blastomeres from early pig embryos at 1- to 8-cell stages, generated from in vitro fertilization (IVF) and parthenogenetic activation (PA), respectively. We analyzed the dynamics of transcriptional regulation (death of maternal transcripts and birth of zygotic transcripts), compared comprehensively the regulation of gene expression between biparental and maternal-only embryos, and provided reference resources for future functional investigation on the key genes and signaling pathways involved.

## Materials and methods

### Ethics statement

All experimental materials and procedures were reviewed and approved by the Animal Care Commission and Ethics Committee of the Yangtze University, Hubei, China. All methods were performed in accordance with the approved guidelines and regulations. Unless specified, all reagents used in this study were purchased from Sigma-Aldrich. Plastic material was bought from Corning.

### In vitro maturation of pig oocytes

Gilt ovaries were obtained from a local slaughterhouse, and transferred to the laboratory within 2 h in sterile 0.9% sodium chloride containing penicillin and streptomycin and maintained at 28–32 °C. Follicular fluids were aspirated from antral follicles (3-5 mm diameter) using an 18-gauge needle attached to a 5 ml disposable syringe. Precipitant was washed three times in the HEPES-buffered Tyrode medium (3.2 mM KCl, 114 mM NaCl, 2 mM CaCl_2_·2H_2_O, 0.34 mM Na_2_HPO_4_, 0.5 mM MgCl_2_, 10 mM sodium lactate, 10 mM HEPES, 0.2 mM sodium pyruvate, 12 mM sorbitol, 2 mM NaHCO_3_, 0.1 mM polyvinylalcohol, 1 μM gentamicin). Pig cumulus-oocyte complexes (COCs) with more than three layers of cumulus cells and uniform ooplasm were picked, and washed using the TCM199 maturation medium (Gibco BRL, Grand Island, NY) supplemented with 0.1% PVA, 3.05 mM D-glucose, 0.91 mM sodium pyruvate, 1 µM gentamicin, 0.57 mM cysteine, 0.5 µM luteinizing hormone, 0.5 µM follicle stimulating hormone, 10 nM epidermal growth factor. COCs were in vitro matured in a 24-well plate (50 COCs in one well, with 500 μl maturation medium and covered by mineral oil). After maturation for 44 h in an incubator (39 °C, 5% CO_2_, and saturated humid air), cumulus cells were stripped off via vortexing in 0.1% hyaluronidase solution in HEPES-buffered Tyrode medium containing 0.01% PVA.

### In vitro fertilization and parthenogenetic activation

MII oocytes were used for in vitro fertilization (IVF) or parthenogenetical activation (PA). For IVF, 25–30 oocytes were put in 50 µl droplets of IVF medium (modified Tris-buffered medium with 113.1 mM NaCl, 3 mM KCl, 7.5 mM CaCl2, 11 mM glucose, 20 mM Tris, 2 mM caffeine, 5 mM sodium pyruvate, and 2 mM BSA). Fresh semen was washed using IVF medium and diluted to 5 × 10^5^ sperms/mL^22^. The semen suspension (50µL) was added into the droplets, and co-incubated for 4 h (39 °C, 5% CO_2_) (end of incubation considered as 0 h for IVF). For PA, oocytes were first placed in the activation medium (0.28 M mannitol, 0.1 mM CaCl_2_·2H_2_O, 0.1 mM MgCl_2_, 1 mM BSA, 0.5 mM HEPES) to reach balance, and then stimulated for 30 μs using two direct pulses of 1.2 kV/cm on the Electrocell Manipulator (BTX830, USA) (marked as 0 h for PA). Then, oocytes were incubated for 4 h in the porcine zygote medium 3 (PZM-3) with 2.5 mM 6-dimethylaminopurine and 5 μM cytochalasin B in the incubator (39 °C, 5% CO_2_ and saturated humid air)^23^. After IVF or activation, 50 zygotes or activated embryos were cultured in 500μL fresh PZM-3 medium covered with mineral oil in the incubator (39 °C, 5% CO_2_ in air atmosphere) for 7 days. The cleavage and the blastocyst formation were recorded at 48 h and 168 h, respectively. Cell numbers of blastocysts were counted after Hoechst33342 staining. Each experiment was repeated at least three times, and data were displayed as means ± standard error of the mean (SEM).

### Single embryo/blastomere preparation

Zygotes or parthenotes were collected from 1-cell (19 h), and blastomeres were biopsied from 2-cell (27 h), 4-cell (48 h) and 8-cell (72 h) IVF and PA embryos, respectively. Acquired embryos were washed three times using 1% bovine serum albumin (BSA) in Dulbecco’s phosphate saline (DPBS), and the zona pellucida was removed using Tyrode medium acidic with HCl. Embryos were treated using 0.05% trypsin in 1% BSA/DPBS for 40 to 60 min, and then physically separated into single blastomeres by repeatedly sucking and blowing, using a fine glass pipette with appropriate diameter. Separated single blastomeres were washed three times with 1% BSA in DPBS, immediately transferred into the lysis buffer in 200μL eppendorf tube, and put into a −80 °C freezer until use.

### Library construction and single cell RNA sequencing

The single cell samples were reversely transcribed and amplified using Ansuper-seq, developed and adapted from a previously published method^24^. Briefly, in this reaction system, specific primers were designed and used to reversely transcribe the first cDNA strand based on templates of transcripts with or without polyA tails. Then, at the end of the first cDNA strand, polyA tails were extensively synthesized and attached, and the second cDNA strand was synthesized using primers binding to the polyA tail. Finally, cDNAs were PCR amplified to obtain enough material for sequencing library construction. To construct the library, cDNA products were fragmented by the Bioruptor®Sonication System (Diagenode Inc.), complemented the terminal sequences, added adenosine at the 3’-terminal, put on sequencing adapters, selected specific size of fragments, and amplified by PCR. The final libraries were evaluated for quality by checking the concentration and fragment distribution, and were submitted for 150 bp paired-end sequencing by an Illumina Hiseq X-Ten platform. All sequencing work was done by the Annoroad Gene Technology Company.

### Read mapping and expression quantification

Raw reads were processed into clean reads by removing low quality and adaptor-containing reads. Then, clean reads were mapped onto the pig reference genome (Sscrofa11.1) by Hisat2^25^. After assessing sequence saturation and read distribution on the reference genome, transcripts were assembled by StringTie^26^, counts were numbered by HTSeq^27^, and FPKM (Fragments Per Kilobase Millon Mapped Reads) were calculated. Sex of single cells was determined, by calculating total FPKM of all genes on the Y chromosome (When ≥ 10, it was regarded as male; whereas ≤ 10, considered as a female)^13^. Differentially expressed genes (DEGs) between two groups were identified using DEseq2^28^. A gene was considered significant if the Benjamini and Hochberg-adjusted P value (Padj.) was < 0.05 and the |log_2_ (Fold change)|≥ 1. ClusterProfiler^29^ in the R software (www.R-project.org/) was used for gene enrichment analysis, Gene Ontology (GO) and KEGG pathways (Kyoto Encyclopedia of Genes and Genomes, www.kegg.jp/kegg/kegg1.html) ^30^, respectively (P < 0.05 defined as significant enrichment). To compare the expression levels of pig in vitro early embryos with in vivo derived embryos, the FPKM of porcine in vivo embryos generated via scRNA-seq was directly downloaded from the published report^13^.

### Significance analysis on important genes and pathways

Based on the biological significance of gene involvement in oocyte maturation and early embryo development (MZT and ZGA), we selected important genes in DNA/histone methylation and transcription factors, and analyzed their expression patterns in our samples.

## Results

### scRNA-seq overview

**Figure 1:**
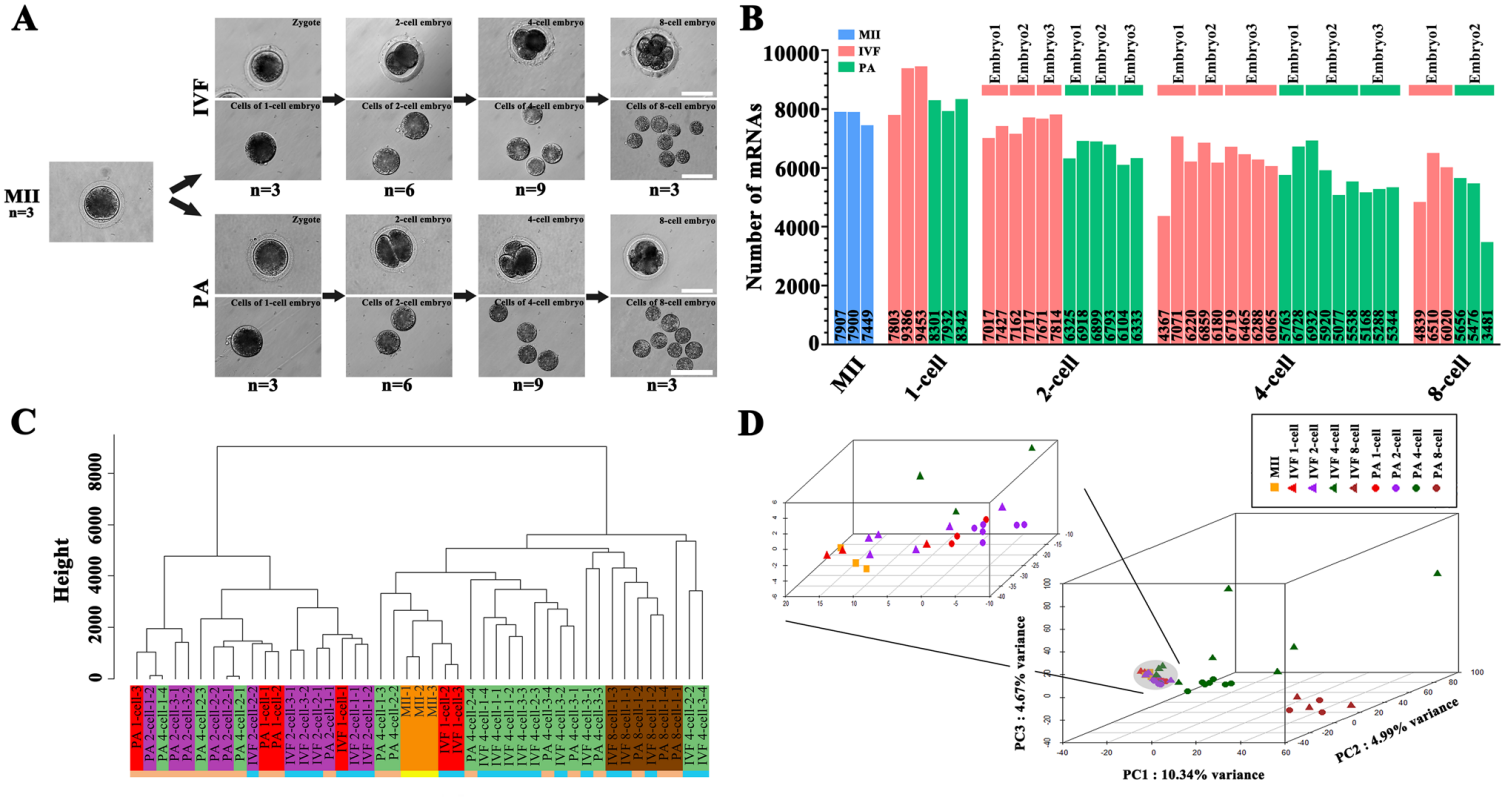
scRNA-seq of porcine mature oocytes and early embryos. **A** Porcine MII oocytes and early embryos were collected (details as in Materials and Methods). Scale bars: 100 μm. **B** Number of expressed mRNAs at different developmental stages. **C** Unsupervised hierarchical clustering. Different colors indicate different developmental stages. **D** PCA analysis. Different colors and shapes indicate different developmental stages and lineages.

To study the dynamic changes of gene expression profiles during MZT process in pig early embryos, we collected and sequenced a total of 42 single cells, 21 each for in vitro fertilization (IVF) and parthenogenetic activation (PA) embryo samples (3 zygotes, 6, 9 and 3 single blastomeres from 2-cell, 4-cell, and 8-cell embryos), respectively (Fig. 1A). All samples were taken from an in vitro culturing and fertilization system established in the lab, to produce porcine early embryos (Figure S1).

Mapping statistics of short reads obtained from scRNA-seq of IVF and PA embryos were summarized in Table S1. Expression levels of mRNAs (FPKM) were calculated for all 42 single cells, and also for mature MII oocytes (n = 3) (Table S2). Generally, the number of detected mRNAs (FPKM > 0) gradually decreased from 1- to 8-cell stages in both IVF and PA groups (Fig. 1B). Based on mRNA expression data, the unsupervised hierarchical clustering and principal-component analysis (PCA) were performed. The unsupervised hierarchical clustering results showed two big "blocks" , the first one containing MII oocytes close to IVF and PA at 1- and 2-cell stages, and the second one with IVF close to PA at 4- and 8-cell stages (Fig. 1C). PCA analysis showed three big blocks: the first one with MII oocytes close to IVF and PA at 1- and 2-cell stages, the second block with IVF and PA at 4-cell stage and third one containing IVF and PA at 8-cell stage (Fig. 1D). Thus, our data indicated that expression differences of mRNAs existed along the developmental stages of porcine early embryos. Furthermore, we characterized the gender of single cells, two of three 4-cell and one 8-cell IVF embryos to be male; all PA embryos to be female (Table S3 and Figure S2). However, expression levels of all genes on the Y chromosome largely differed among different blastomeres within the same male 4- or 8-cell IVF embryo, indicating asymmetric or imbalanced expression.

### Abundantly expressed genes in early embryos

Before focusing on genes significantly differentially expressed between different developmental stages, or between IVF and PA embryos, we first examined those genes expressed the most abundantly, considered to be vital to maintain the basic characteristics of specific cell/tissue/organ. For all 42 samples, top 20 abundantly expressed mRNAs were selected, and we found that many mitochondrion-derived RNAs were highly abundantly expressed in both IVF and PA groups (Table S4), similar to results of in vivo oocytes and fertilized embryos.

**Figure 2:**
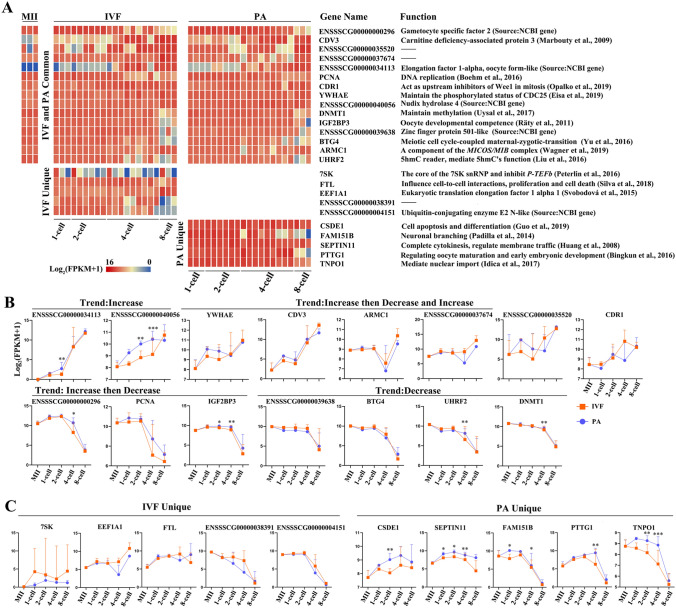
Nuclear mRNAs abundantly expressed (Top 20, starting from the highest average FPKM). **A** Heatmap and functional annotation of top 20 abundantly expressed mRNAs in IVF and PA embryos, including common and specific transcripts to IVF and PA embryo samples. **B** Expression dynamics of common (IVF and PA) mRNAs (mean ± SD). **C** Expression dynamics of unique mRNAs (mean ± SD).

Then, we looked at the top 20 abundantly expressed nuclear mRNAs at each stage of IVF and PA groups (Table S5) (Fig. 2). First, common mRNAs to both IVF and PA embryos (MII to 8-cell) were ranked (Fig. 2A, upper section). Evidences indicate that these genes (CDV3, PCNA, CDR1, YWHAE, DNMT1, IGF2BP3, ARMC1, BTG4, UHRF2, gametocyte-specific factor 1-like) are important in multiple biological processes, including the fertilization and early embryo development (see references in Fig. 2 and Table S4). Second, genes specific to IVF or PA groups were also found, such as FTL, 7SK, ENSSSCG00000038391, ENSSSCG00000004489, ENSSSCG00000004151 in the IVF group, and SEPTIN11, CSDE1, FAM151B, ENSSSCG00000017032 and TNPO1 in the PA group, respectively (Fig. 2A, lower section). In addition, after comparing the expression dynamics of those mRNAs (Fig. 2A) and abundances in the IVF and PA groups (Fig. 2B and 2C), to those of the in vivo group (Figure S3)^13^, we found that some mRNAs (including CDR1, YWHAE, IGF2BP3, gametocyte-specific factor 1-likeand TNPO1) were highly expressed in IVF and/or PA groups, but lowly expressed in vivo. Thus, these data provide an important resource and information to further investigate the involvement of these genes in porcine oocytes and early embryos, both in vitro and in vivo.

### ZGA timing of in vitro porcine embryos

**Figure 3:**
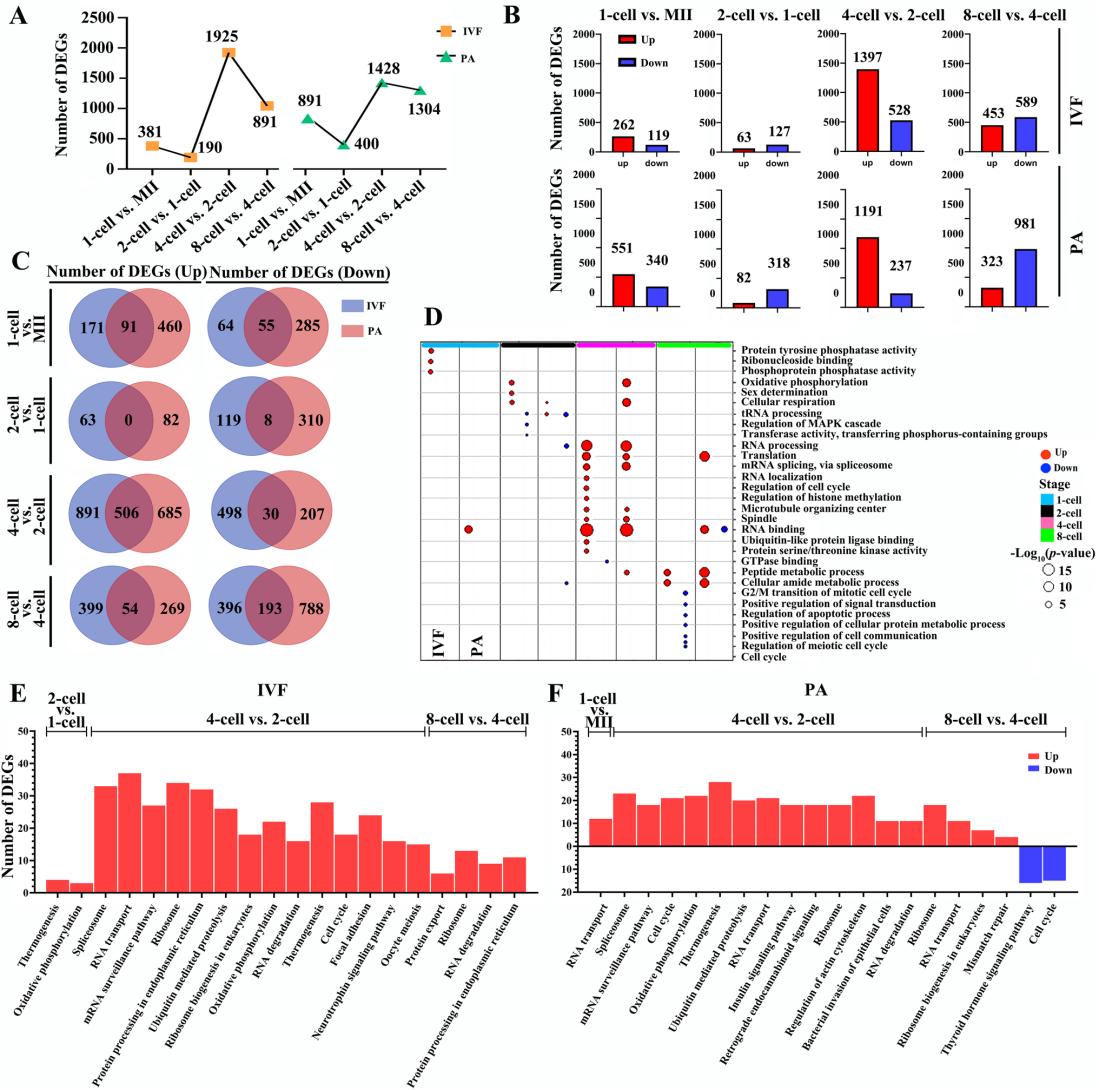
Pairwise analysis of DE mRNAs within pig IVF or PA early embryos. **A** Total number of DE mRNAs. **B** Number of up- and down-regulated mRNAs. **C** Comparison of up- and down-regulated mRNAs. **D** GO analysis. **E** and **F** KEGG (www.kegg.jp/kegg/kegg1.html) pathways. ClusterProfiler in the R software (www.R-project.org/) was used to analyze GO and KEGG enrichment.

Previously, ZGA is determined to happen at the 4-cell to 8-cell embryonic stages for in vivo porcine embryos^13^. To better understand the molecular events of genome activation during MZT (minor and major ZGAs), we first analyzed mRNAs differentially expressed between consecutive developmental stages within IVF and PA groups (Table S6). Between MII oocytes, 1-cell zygotes, and subsequent 2-cell, 4-cell and 8-cell embryos, 262, 63, 1397 and 453 genes were found to be activated (up-regulated) for IVF embryos (fold change ≥ 2 and adjusted P (Padj.) < 0.05), while for the PA embryos, 551, 82, 1191, 323 genes were activated, respectively (Fig. 3A and 3B). Thus, based on the number of genes activated in pig in vitro embryos, initial activation of genome transcription (minor ZGA) could begin as early as 1-cell stage and continue to 2-cell stage. The largest number of genes up-regulated indicated that the major ZGA occurs during the transition from 2-cell to 4-cell stages. For IVF and PA embryos, although minor and major ZGAs occurred at the same stages, and exhibited similar trends, numbers of up-and down-regulated DE mRNAs were different (Fig. 3B). Moreover, at each stage, relatively few of the up- and down-regulated genes were common (Fig. 3C), suggesting bi-parental and maternal-only embryos had specific sets of genes distinctly activated.

In the present study, 119, 127, 528 and 589 mRNAs were significantly down-regulated from MII to 8-cell stage of IVF embryos (fold change < 0.5 and Padj. < 0.05), whereas 340, 318, 237 and 981 genes, in the PA group (Fig. 3B), respectively. Thus, both M-decay and Z-decay occurred in the pig bi-parental and maternal-only early embryos. However, PA embryos had more genes up- and down-regulated from MII oocyte to 2-cell stages (minor ZGA over-activated), but less genes at the 4-cell stage (suggesting incomplete major ZGA). Furthermore, less genes up-regulated but more genes down-regulated from 4- to 8-cell embryonic stage might cause the inactivation of signaling pathways important for embryo development in the PA embryos at the 4-cell major ZGA stage, such as RNA localization, regulation of cell cycle and histone methylation (Fig. 3D; Tables S7 and S8).

In IVF and PA embryos, RNA binding and processing were the main enriched GO pathways at 4-cell stage, while at 8-cell stage, translation and peptide metabolic process were the main pathways (Fig. 3D; Tables S7 and S8). KEGG analysis also showed that RNA transport/degradation, oxidative phosphorylation, cell cycle, thermogenesis and ribosome pathways were enriched in IVF and PA embryos from 2- to 8-cell stage (Fig. 3E and F). In contrast, neurotrophin pathway was up-regulated only at 2- to 4-cell of IVF embryos (Fig. 3E) and insulin pathway was up-regulated only at 2- to 4-cell of PA embryos (Fig. 3F) (Tables S7 and S8).

Therefore, in IVF and PA embryos, maternal and paternal genome could contribute differently to MZT (for both maternal RNA clearance and ZGA), and determine the subsequent successful full-term embryo development.RNA associated pathways could be important to the developmental competence of pig in vitro early embryos.

### Stage-wise comparison between IVF and PA embryos

**Figure 4:**
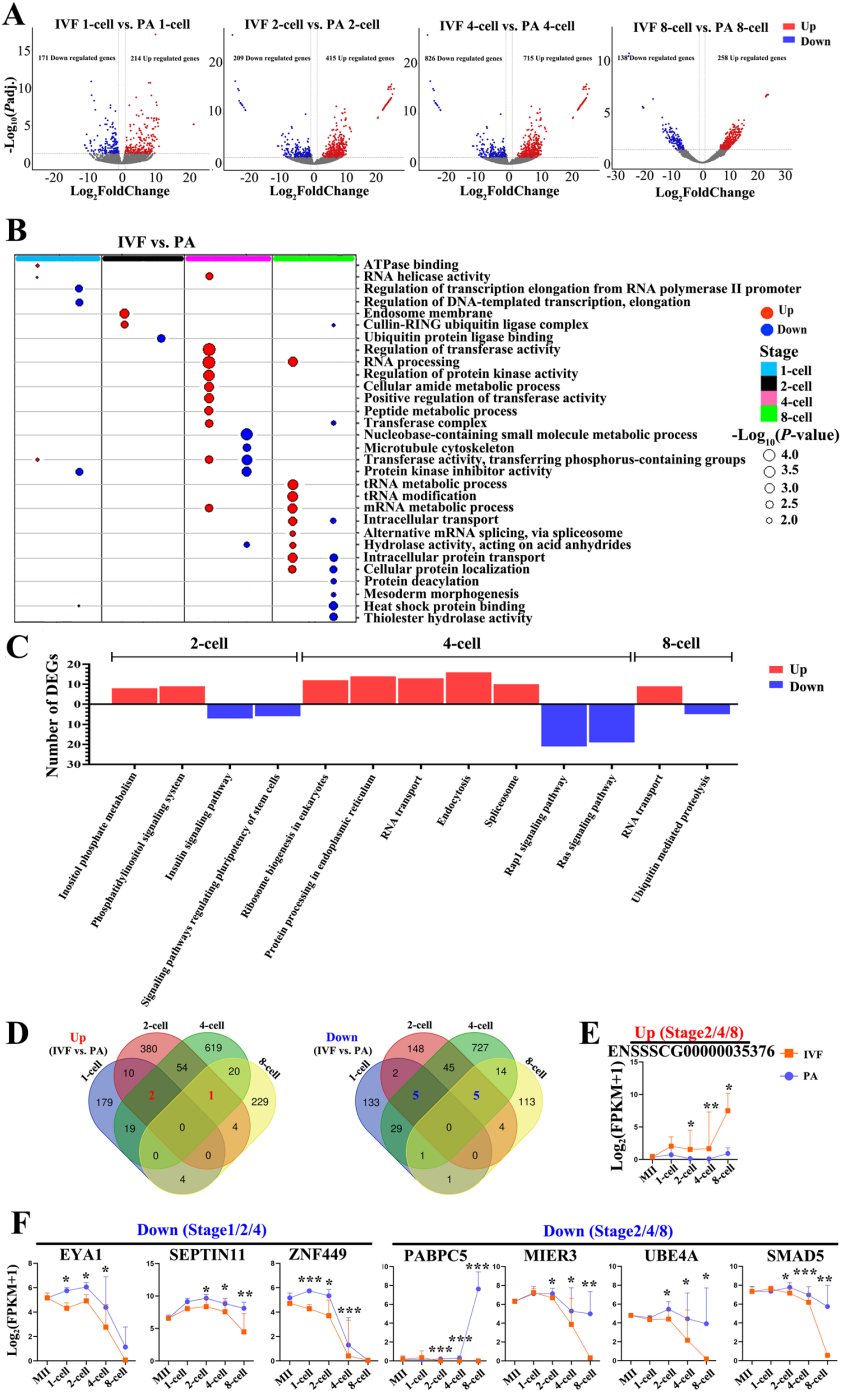
DE mRNAs between IVF and PA. **A** Volcano plots with number of up- and down-regulated mRNAs. **B** and **C** GO processes and KEGG (www.kegg.jp/kegg/kegg1.html) pathways. ClusterProfiler in the R software (www.R-project.org/) was used to analyze GO and KEGG enrichment. **D** VennDiagram to show the common up- and down-regulated mRNAs during 1- to 8-cell stages. **E** ENSSSCG00000035376, one up-regulated mRNA common to 2-, 4- and 8-cell stages. **F** three down-regulated mRNAs (EYA1, SEPTIN11 and ZNF449) common to 1-, 2- and 4-cell stages and other four ones (PABPC5, MIER3, UBE4A and SMAD5) common to 2-, 4- and 8-cell stages.

To further understand the molecular differences between IVF and PA embryos, we compared the differentially expressed genes between each developmental stage (from 1- to 8-cell embryos) (Table S9). We found a large number of differentially expressed genes at all embryonic stages, and especially at the 4-cell stage, more genes were up- and down-regulated (Fig. 4A). In IVF embryos, GO analysis found up-regulated pathways (transferase activity, RNA processing and protein kinase activity at 4-cell stage; and tRNA metabolic process, tRNA modification and mRNA metabolic process at 8-cell stage), and down-regulated pathways (small molecule metabolic process and protein kinase inhibitor activity at 4-cell stage; heat shock protein binding and thiolester hydrolase activity at 8-cell stage) (Fig. 4B; Table S10). Additional KEGG analysis showed that RNA transport, protein processing in endoplasmic reticulum and ribosome biogenesis pathways at 4- and 8-cell stage were up-regulated, and in contrast, insulin, pluripotency, Rap1, Ras and ubiquitin mediated proteolysis pathways at 2- and 8-cell stages were down-regulated (Fig. 4C; Table S10). Moreover, most of up- or down-regulated genes were stage-specific (Fig. 4D). We selected some common genes and analyzed their expression profiles, such as one up-regulated gene, ENSSSCG00000035376 (common to 2-, 4- and 8-cell embryos) (Fig. 4E), and down-regulated genes, EYA1, SEPTIN11 and ZNF449 (common to 1-, 2- and 4-cell embryos), as well as PABPC5, MIER3, UBE4A and SMAD5 (common to 2-, 4- and 8-cell embryos) (Fig. 4F). Thus, stage-wise comparison found genes and signaling pathways specific to bi-parental and maternal-only embryos.

### Mitochondrial activation

**Figure 5:**
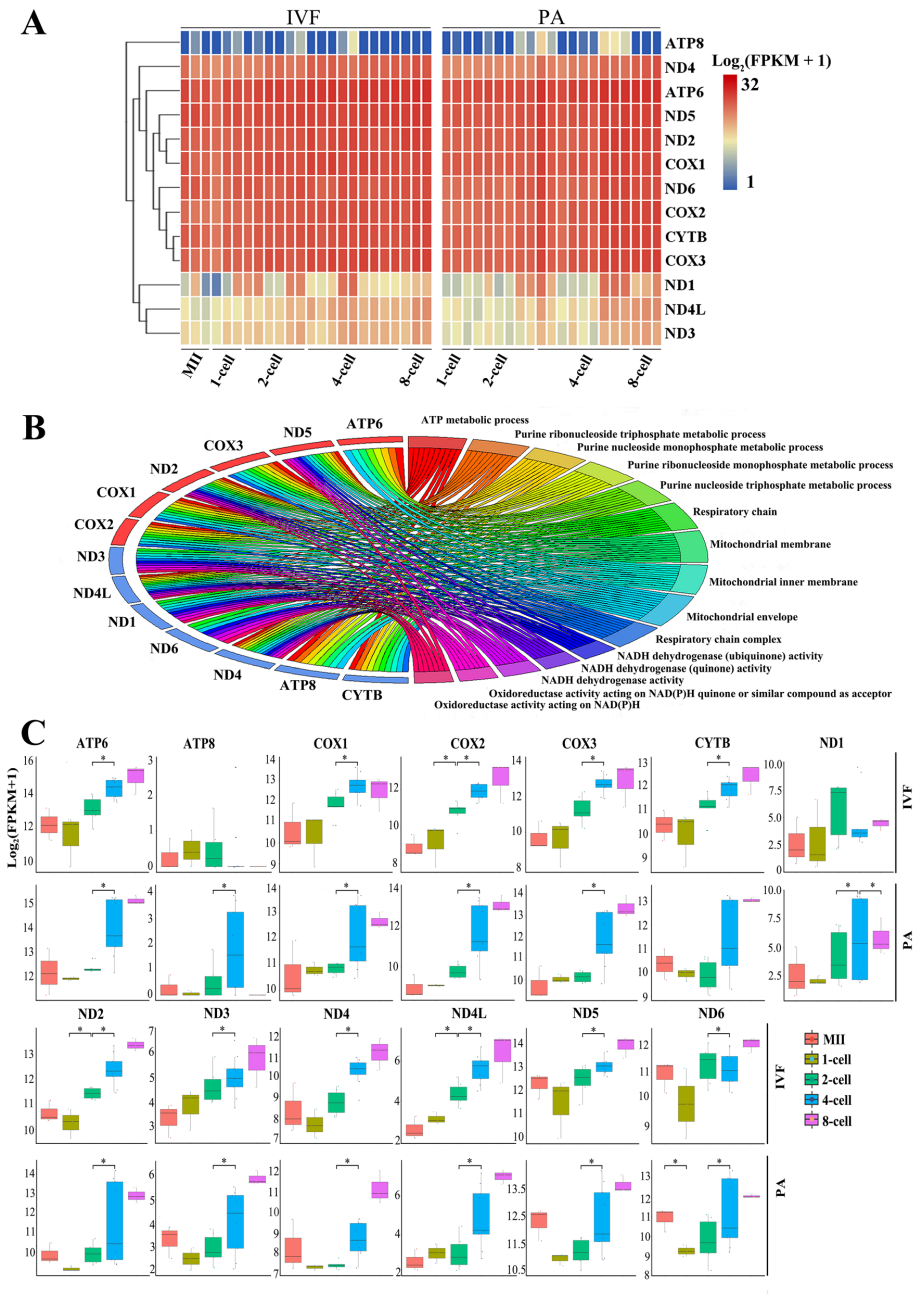
Mitochondrial mRNAs. **A** Heatmap of 13 mitochondrial mRNAs. **B** GO processes. **C**, Expression dynamics.

It is well recognized that mitochondria are important to the development of early embryo^31^. In the present study, in both IVF and PA embryos, mitochondrial genes enriched in metabolic pathways (NADH activity and ATP metabolism) were found to be highly abundantly expressed, including protein-coding mRNAs (ATP6, ND2/4/5/6, COX1/2/3 and CYTB) (Fig. 5A and B) (Table S11). Along with the development of early embryos (from 1- to 8-cell) in both IVF and PA groups, expression levels of most protein-coding genes in mitochondria increased, suggesting that in vitro development of pig early embryos requires high energy input (Fig. 5C). Moreover, some of mitochondria derived protein-coding mRNAs showed significant difference between two adjacent stages, mostly during the transition from 2-cell to 4-cell embryos. Mitochondrial mRNAs of IVF and PA embryos presented similar changing patterns. However, when comparing embryos developed in vitro to in vivo^13^, ATP8 level was significantly lower in the in vitro IVF and PA embryos (Fig. 5A and C), but extremely higher in the in vivo embryos (Figure S4). Similarly, ND1, ND3 and ND4L were also lower in IVF and PA embryos than those in vivo. Several other mitochondrial mRNAs also showed dynamic expression patterns between in vitro and in vivo embryos. Thus, embryos developed in vitro could require additional energy input for successful development due to incomplete mitochondrial function.

### Dynamics of genes important for embryo development

**Figure 6:**
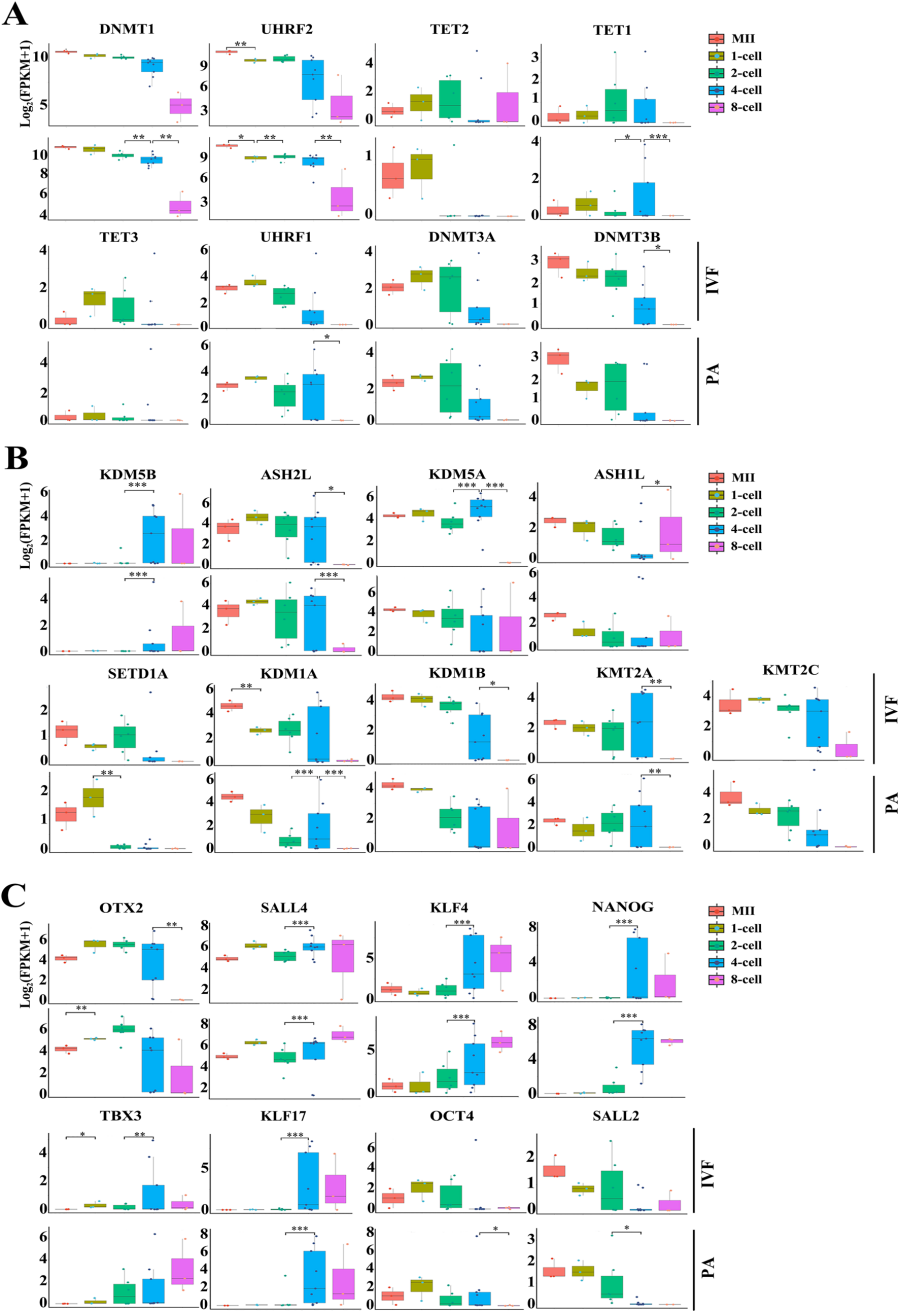
Transcription dynamics of genes related to DNA methylation, H3K4 methylation and transcription factors. Expression dynamics of genes related to DNA methylation (**A**), H3K4 methylation (**B**) and transcriptional factors (**C**).

Since successful genome (de)methylation is the key process to the developmental reprogramming of early embryos, we evaluated also the dynamic expression pattern of DNA and H3K4 methylation related genes. In in vitro developed early embryos, DNMT1 and UHRF2 were highly expressed (Fig. 6A), other genes related to DNA methylation showed relatively lower expression (Fig. 6A; Table S12). In in vivo embryos, DNMT1 and UHRF2 were also highly expressed, but DNMT1 was absent in oocytes collected in vivo (Figure S5 and Table S12). As for expression levels of TET family genes (TET1, TET2 and TET3), they all showed lower expression in in vitro IVF and PA embryos (Fig. 6A), as well as in in vivo embryos (Figure S5).

We analyzed further the expression pattern and dynamics of genes related to histone methylation at lysine 4 of histone 3 (H3K4me). Most of them showed low expression levels, whereas ASH2L and KDM5A were relatively high (Fig. 6B; Table S11). However, most of H3K4me-related genes had significant expression changes when transitioning from 4- to 8-cell embryos (Padj < 0.05 and |log_2_ (Foldchange)|≥ 1) (Fig. 6B). In contrast, overall expression of H3K4me-related genes was higher in in vivo embryos (Figure S5). Taken together, these data indicate that environmental or epigenetic differences between in vitro and in vivo embryos could alter the gene expression of DNA/histone modifications during MZT of pig early embryos, potentially affecting developmental competence.

In an attempt to dissect the regulatory gene network potentially underlying early embryo development, and also ZGA dynamics, we analyzed expression profiles of important transcription factors (TFs) in both IVF and PA embryos (Fig. 6C; Table S12). OTX2 expressed abundantly in MII oocytes and early embryos (1- to 4-cell), but decreased at 8-cell stage, however, SALL4 showed high expression through all developmental stages (Fig. 6C). We found that a common set of TFs (SALL4, NANOG, KLF4, and KLF17) were all significantly differentially expressed at the major ZGA stage (4-cell/2-cell) (Fig. 6C). In addition, NANOG, KLF4 and KLF17 showed similar expression patterns. We found that expression differences of key TFs (OTX2, OCT4 and SALL2) did exist between IVF and PA embryos. However, compared to in vivo embryos (Figure S5), overall expression levels of TFs were lower in IVF and PA embryos, especially for OCT4, KLF7, SOX2 and SOX15. Thus, these data indicate that transcriptional regulatory network composed of key TFs could be fundamental to the in vitro development of pig early embryos.

## Discussion

After fertilization, oocyte and sperm unite together, and become a totipotent zygote. However, the zygotic genome is transcriptionally quiescent, providing the time window required to be reprogrammed into a totipotent state. Then, MZT (including maternal RNA decay and ZGA) appears, during which the zygotic genome gradually restarts transcription, and maternal gene products deposited in the oocytes are replaced^32,33^. MZT is essential for embryogenesis and successful full-term development, but its control dynamics is still obscure in pigs. In the present study, we analyzed the transcriptome using scRNA-seq on mature oocytes, zygotes and embryonic ones (2- to 8-cell) collected from pig IVF and PA early embryos, to systematically characterize the MZT-associated dynamics of transcripts.

Based on transcripts detected in all samples at different embryo stages, we found that the number of expressed mRNAs varied in different type of embryos, different embryonic stages, different embryos at the same stage, and different blastomeres from the same embryo, reflecting the heterogeneity of single embryo/blastomere. However, the overall number of expressed transcripts decreased from 1- to 8-cell embryonic stage, indicating that majority of transcripts decayed during MZT transition. scRNA-seq helped us to identify the most abundant transcripts in pig oocytes and early IVF/PA embryos, including protein-coding RNAs from both mitochondrial and nuclear genomes. Mitochondria are maternally inherited in mammals^34^, playing an essential role in the regulation of oocyte maturation and early embryo development via oxidative phosphorylation to provide energy^31,35^. Mitochondrial mRNAs (except ATP8) gradually increase from MII oocytes to 8-cell embryos in both pig IVF and PA groups, suggesting that active transcription of mitochondrial genome is needed to supply the basic energy requirement for MZT during early embryo development. This is supported by the observation that mitochondrial DNA lacks 5mC methylation in mouse oocytes and early embryos^36^. However, in contrast to in vivo embryos, we found the lower expression of ATP8 in in vitro embryos (both IVF and PA). This difference might contribute to the poor quality and developmental competence of early embryos, since ATP8 plays an important role in ATP processing and the respiration chain^37^. Supportive evidences also show that treatment of maturing mouse oocytes by carbonyl cyanide p-(tri-fluromethoxy) phenyl-hydrazone (FCCP) to reduce oxidative phosphorylation could decrease ATP content, and thereby negatively impact oocyte quality and embryo development^38^. In addition to mitochondrial transcripts, abundant nuclear transcripts were also identified, some of which have been previously confirmed to be vital for biological processes, such as BTG4 for MZT^39^, DNMT1 and UHRF2 for DNA methylation^40^, CDR1^41^ and YWHAE^42^ for mitotic cell cycle, PCNA for DNA replication^43^, IGF2BP3 for embryo quality^44^, and ARMC1 for mitochondrial function^45^. Moreover, the remaining abundant transcripts (ENSSSCG00000000296, CDV3, ENSSSCG0000000035520, ENSSSCG0000000037674, ENSSSCG0000000034113, ENSSSCG0000000040056 and ENSSSCG0000000039638) still await further investigation, to uncover their function in the maintenance and regulation of early embryo development.

Transcriptome differences along developmental stages for pig IVF or PA groups were analyzed in the present study. First, genomes of 1-cell zygotes were actively transcribed, suggesting minor ZGA occurring as early as 1-cell stage, similar to mice^8^. Second, a large number of transcripts increased from 2- to 4-cell stage in both IVF and PA groups, suggesting that major ZGA is at 4-cell stage. Third, maternal RNA decay (both M- and Z-decay) occurred actively from 1- to 8-cell stages, and majority of transcripts were degraded from 4- to 8-cell stages. Fourth, most of up- or down-regulated genes were different between IVF and PA groups, suggesting that molecular differences in MZT regulatory dynamics exist. Fifth, the lower number of common DEGs indicated different signaling pathways were involved in IVF and PA embryo development. Sixth, pairwise comparison between IVF and PA embryos showed that several hundreds of up- and down-regulated DEGs at each of 1- to 8-cell stages (largest number of DEGs at 4-cell stage) were enriched in multiple signaling pathways. Collectively, these data indicate that DEGs and signaling pathways related to MZT are different for early embryos derived from bi-parental and maternal-only genome background in pigs.

Epigenetic modification of chromatins could affect MZT, and regulate early embryo development^46^, such as DNA methylation^7^ and H3K4 methylation^47,48^. Maternal transcriptional factors are required for ZGA during MZT^49^, and spatio-temporal expression of transcriptional factors accompanies MZT during embryogenesis^50^. In the present study, scRNA-seq demonstrated that for in vitro and in vivo embryos, expression of chromatin factors and transcriptional factors had large differences, suggesting that the in vitro embryo culture system could significantly alter the gene expression and influence the developmental ability. For DNA methylation factors (UHRF1/2 and DNMT1/3A/3B), expression levels and changing trends were very different between in vitro and in vivo embryos, indicating that DNA methylation difference caused by these factors might possibly affect the transcription integrity^51^ and preimplantation development^52^. Similar results for factors related to H3K4 methylation and transcription were also found to be different between in vitro and in vivo groups. These epigenetic factors might contribute to the regulation of embryo transcriptome and development at specific stages.

## Conclusion

Transcriptome dynamics for in vitro pig embryo development were systematically catalogued, and key genes and signal pathways were identified for MZT (ZGA and maternal RNA decay), which provides vital information and resources for further investigation.

## Supplementary Information

Supplementary Figures and Supplementary Table S1 and S3

Supplementary Table S2

Supplementary Table S4

Supplementary Table S5

Supplementary Table S6

Supplementary Table S7

Supplementary Table S8

Supplementary Table S9

Supplementary Table S10

Supplementary Table S11

Supplementary Table S12

## Data Availability

All obtained scRNA-seq data in the present study have been deposited in NCBI GEO repository (http://www.ncbi.nlm.nih.gov/geo/) (GSE164812). For in vitro matured MII oocytes (n = 3), our scRNA-seq data were previously deposited (GSE160334).
