# Supplementary material for: Single cell RNA-seq reveals genes vital to in vitro fertilized embryos and parthenotes in pigs
Source: Sci Rep. 2021 Jul 13;11:14393. doi: 10.1038/s41598-021-93904-3 (PMC8277874; doi:10.1038/s41598-021-93904-3)
Supplement: Supplementary file 1 — Supplementary Figures and Supplementary Table S1 and S3 [file 41598_2021_93904_MOESM1_ESM.pdf]

Single cell RNA-seq reveals genes vital to *in vitro* fertilized embryos and parthenotes in pigs

Zhi-Qiang Du[1,2#], Hao Liang[1#], Xiao-Man Liu[1], Yun-Hua Liu[2], Chonglong Wang[3], Cai-Xia Yang[1,2*]

Supplemental Tables

Table S1. Mapping statistics of scRNA-seq in IVF and PA embryos.

	#Library	MII-1	MII-2	MII-3	1-cell-1	1-cell-2	1-cell-3	2-cell-1-1	2-cell-1-2	2-cell-2-1	2-cell-2-2	2-cell-3-1	2-cell-3-2
IVF	Total Reads	94,669,202	78,831,566	75,662,344	66,882,442	86,937,340	81,559,278	56,431,708	77,368,416	92,949,604	97,954,362	80,920,488	84,817,268
	Mapped Reads	91,236,050	75,740,247	73,035,506	62,582,603	83,588,093	78,279,592	51,284,831	74,191,291	88,285,911	93,211,184	77,553,715	81,183,595
	Mapping Rate	0.9637	0.9608	0.9653	0.9357	0.9615	0.9598	0.9088	0.9589	0.9498	0.9516	0.9584	0.9572
	UnMapped Reads	3,433,152	3,091,319	2,626,838	4,299,839	3,349,247	3,279,686	5,146,877	3,177,125	4,663,693	4,743,178	3,366,773	3,633,673
	MultiMap Reads	662,821	548,584	465,510	475,869	640,068	579,685	414,092	616,613	603,577	649,542	613,388	609,310
	MultiMap Rate	0.007	0.007	0.0062	0.0071	0.0074	0.0071	0.0073	0.008	0.0065	0.0066	0.0076	0.0072

	#Library	4-cell-1-1	4-cell-1-2	4-cell-1-4	4-cell-2-2	4-cell-2-3	4-cell-3-1	4-cell-3-2	4-cell-3-3	4-cell-3-4	8-cell-1-1	8-cell-1-2	8-cell-1-3
IVF	Total Reads	49,620,720	84,203,192	83,454,508	75,899,948	88,470,236	75,107,110	91,185,046	97,599,502	104,815,104	36,094,630	89,278,520	77,476,742
	Mapped Reads	46,085,478	80,122,969	79,180,378	72,771,519	84,082,499	71,248,984	85,826,916	92,511,832	99,027,815	33,341,622	85,138,321	72,698,303
	Mapping Rate	0.9288	0.9515	0.9488	0.9588	0.9504	0.9486	0.9412	0.9479	0.9448	0.9237	0.9536	0.9383
	UnMapped Reads	3,535,242	4,080,223	4,274,130	3,128,429	4,387,737	3,858,126	5,358,130	5,087,670	5,787,289	2,753,008	4,140,199	4,778,439
	MultiMap Reads	390,678	681,289	884,400	1,838,196	1,448,038	1,646,911	1,204,898	746,029	3,328,876	1,071,457	2,267,941	3,090,249
	MultiMap Rate	0.0079	0.0081	0.0106	0.0242	0.0164	0.0219	0.0132	0.0076	0.0318	0.0297	0.0254	0.0399

	#Library	1-cell-1	1-cell-2	1-cell-3	2-cell-1-1	2-cell-1-2	2-cell-2-1	2-cell-2-2	2-cell-3-1	2-cell-3-2	4-cell-1-3	4-cell-1-4	4-cell-2-1
PA	Total Reads	102,292,004	99,599,690	80,264,286	77,466,640	94,312,774	78,842,380	101,242,270	90,395,050	88,782,540	82,045,538	94,847,912	82,683,170
	Mapped Reads	97,489,378	94,714,022	76,828,842	74,283,712	90,578,305	74,700,794	95,824,829	85,947,494	84,794,851	78,206,890	90,934,329	78,233,511
	Mapping Rate	0.953	0.9509	0.9572	0.9589	0.9604	0.9475	0.9465	0.9508	0.9551	0.9532	0.9587	0.9462
	UnMapped Reads	4,802,626	4,885,668	3,435,444	3,182,928	3,734,469	4,141,586	5,417,441	4,447,556	3,987,689	3,838,648	3,913,583	4,449,659
	MultiMap Reads	649,292	611,590	526,715	504,834	611,905	506,766	692,565	630,513	721,960	1,299,138	625,403	540,316
	MultiMap Rate	0.0063	0.0061	0.0066	0.0065	0.0065	0.0064	0.0068	0.007	0.0081	0.0158	0.0066	0.0065

	#Library	4-cell-2-2	4-cell-2-3	4-cell-2-4	4-cell-3-1	4-cell-3-3	4-cell-3-4	8-cell-1-1	8-cell-1-2	8-cell-1-4
PA	Total Reads	86,589,260	72,742,644	69,153,602	64,152,784	79,537,826	71,625,456	97,665,026	70,791,196	75,585,662
	Mapped Reads	82,129,734	69,217,003	65,491,891	60,700,039	75,840,246	67,868,408	93,705,282	67,802,387	72,070,447
	Mapping Rate	0.9485	0.9515	0.947	0.9462	0.9535	0.9475	0.9595	0.9578	0.9535
	UnMapped Reads	4,459,526	3,525,641	3,661,711	3,452,745	3,697,580	3,757,048	3,959,744	2,988,809	3,515,215
	MultiMap Reads	1,158,832	702,598	1,090,946	892,330	1,337,965	688,878	1,446,922	1,152,064	1,656,297
	MultiMap Rate	0.0134	0.0097	0.0158	0.0139	0.0168	0.0096	0.0148	0.0163	0.0219

Table S3. The sex of samples and embryos for scRNA-seq.

	Samples		Embryos	
	**Female**	**Male**	**Female**	**Male**
**MII**	3	0	3	0
**IVF1**	3	0	3	0
**IVF2**	6	0	3	0
**IVF4**	6	3	1	2
**IVF8**	0	3	0	1
**PA1**	3	0	3	0
**PA2**	6	0	3	0
**PA4**	9	0	3	0
**PA8**	3	0	1	0

Supplementary figures

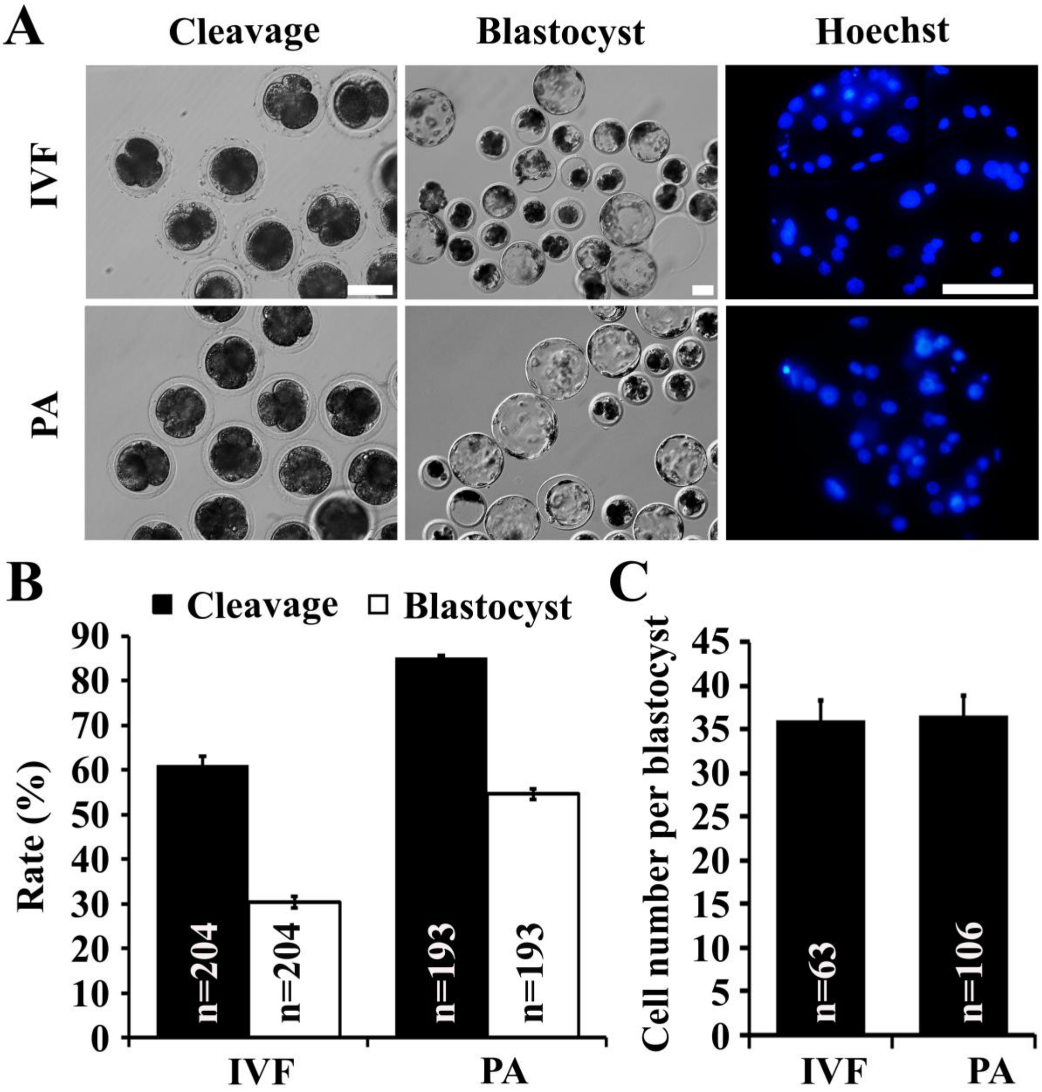

Figure S1. Embryos produced for scRNA-seq. A, Morphology of cleaved embryos (first column), blastocysts (second column) and Hoechst33342 stained blastocysts (third column), derived from IVF and PA, respectively. Scale bars: 100μm. B, Rates of cleaved and blastocyst embryos, respectively. C, Average cell number of blastocysts.

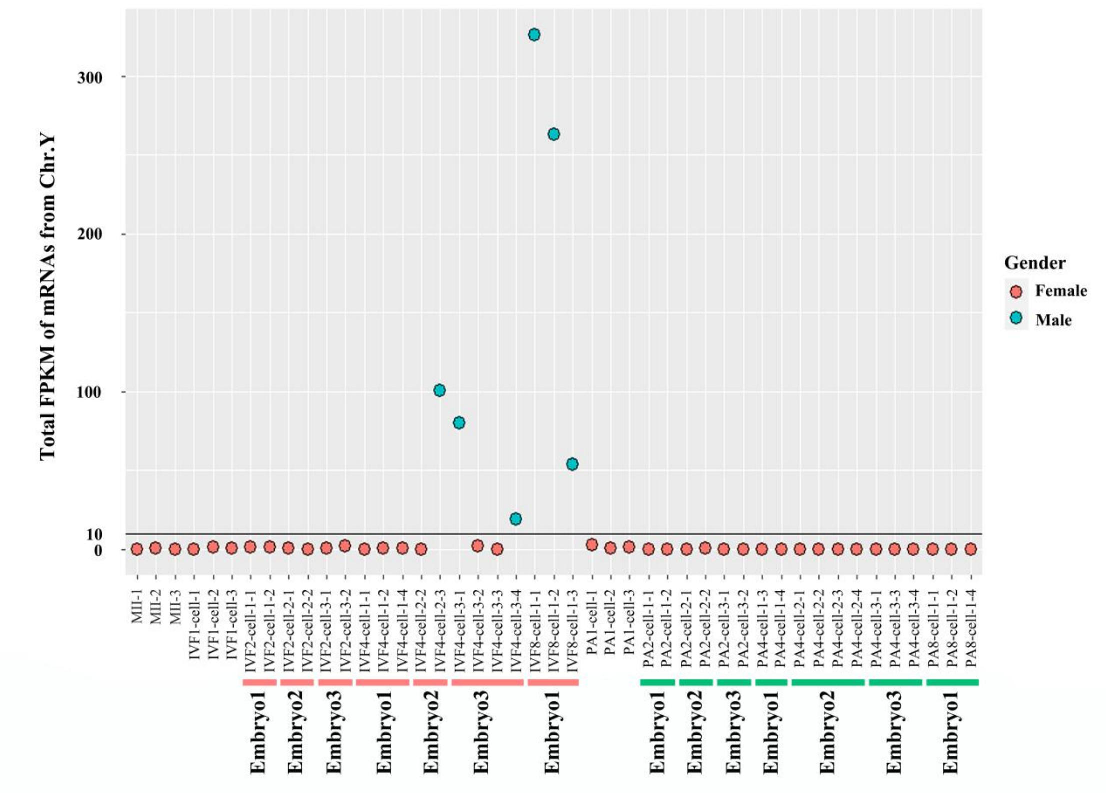

Figure S2. Gender of blastomeres detected in the present study. Each blastomere from pig early IVF and PA embryos was sexed based on the total FPKM of genes on the Y chromosome.

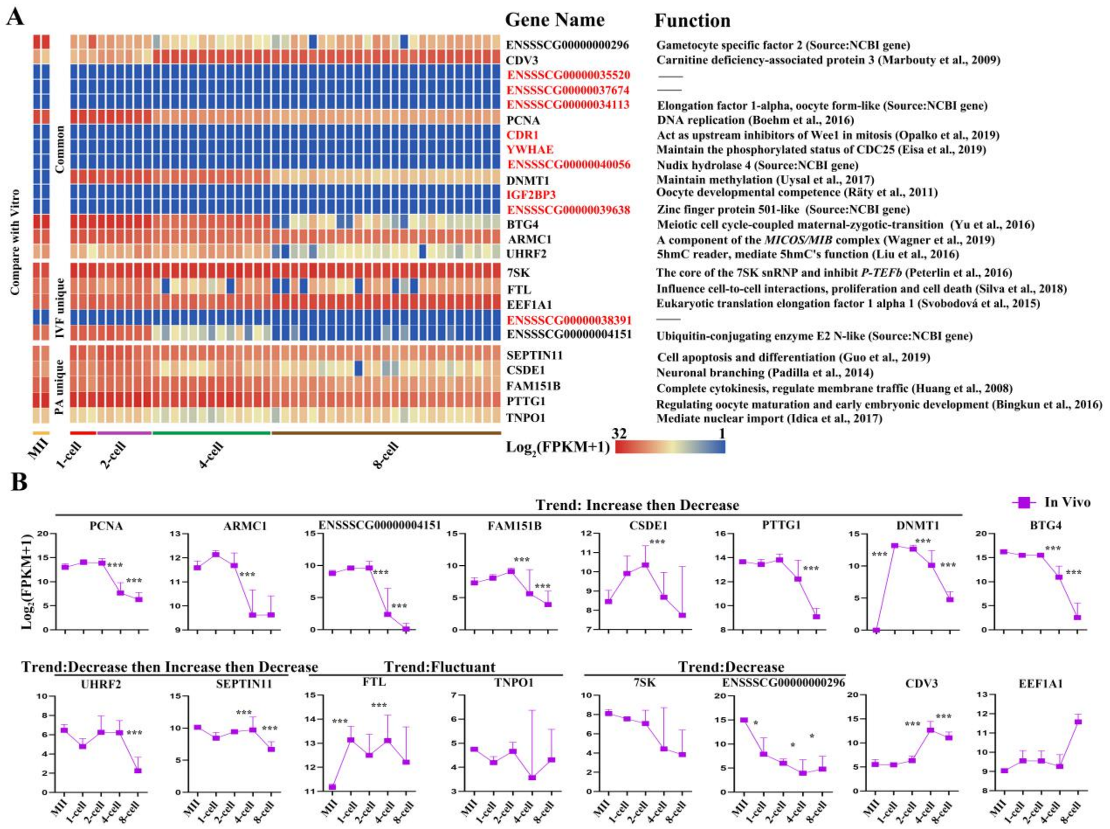

Figure S3. In vivo expression levels of nuclear mRNAs that were listed in the top 20 of pig in vitro embryos (extracted from Table S1 in (Kong et al., 2020))**.** A, Heatmap and functional annotation, including mRNAs common, or specific to IVF or PA groups. B, Expression dynamics of mRNAs.

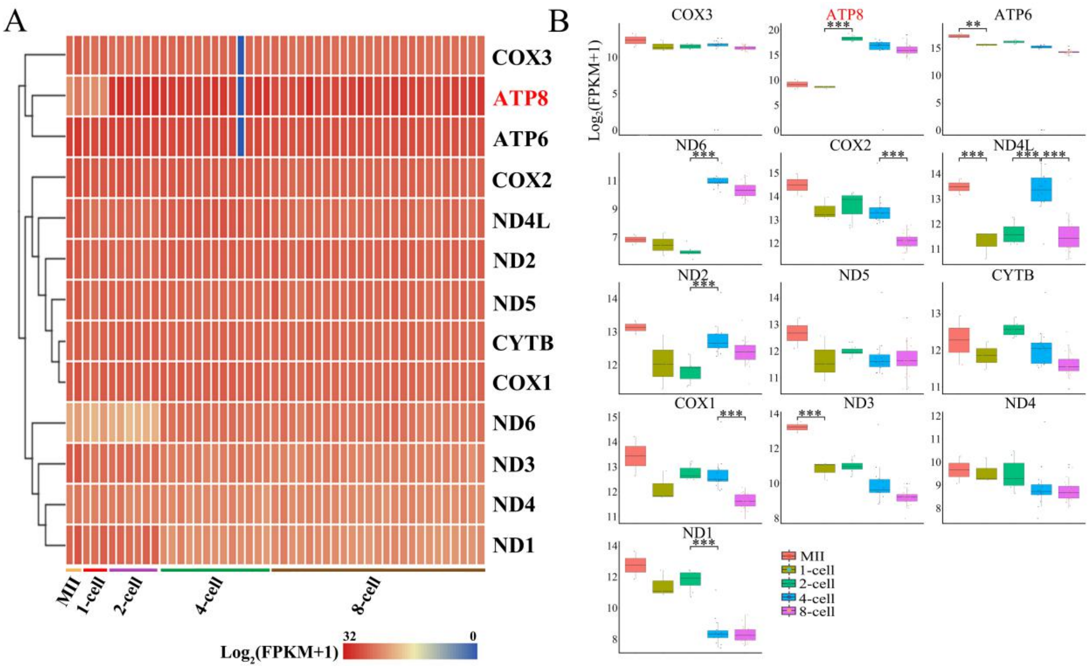

Figure S4. Abundance of mitochondrial mRNAs in pig in vivo embryos (extracted from Table S1 in (Kong et al., 2020))**.** A, Heatmap of mitochondrial mRNAs. B, Expression dynamics of mitochondrial mRNAs.

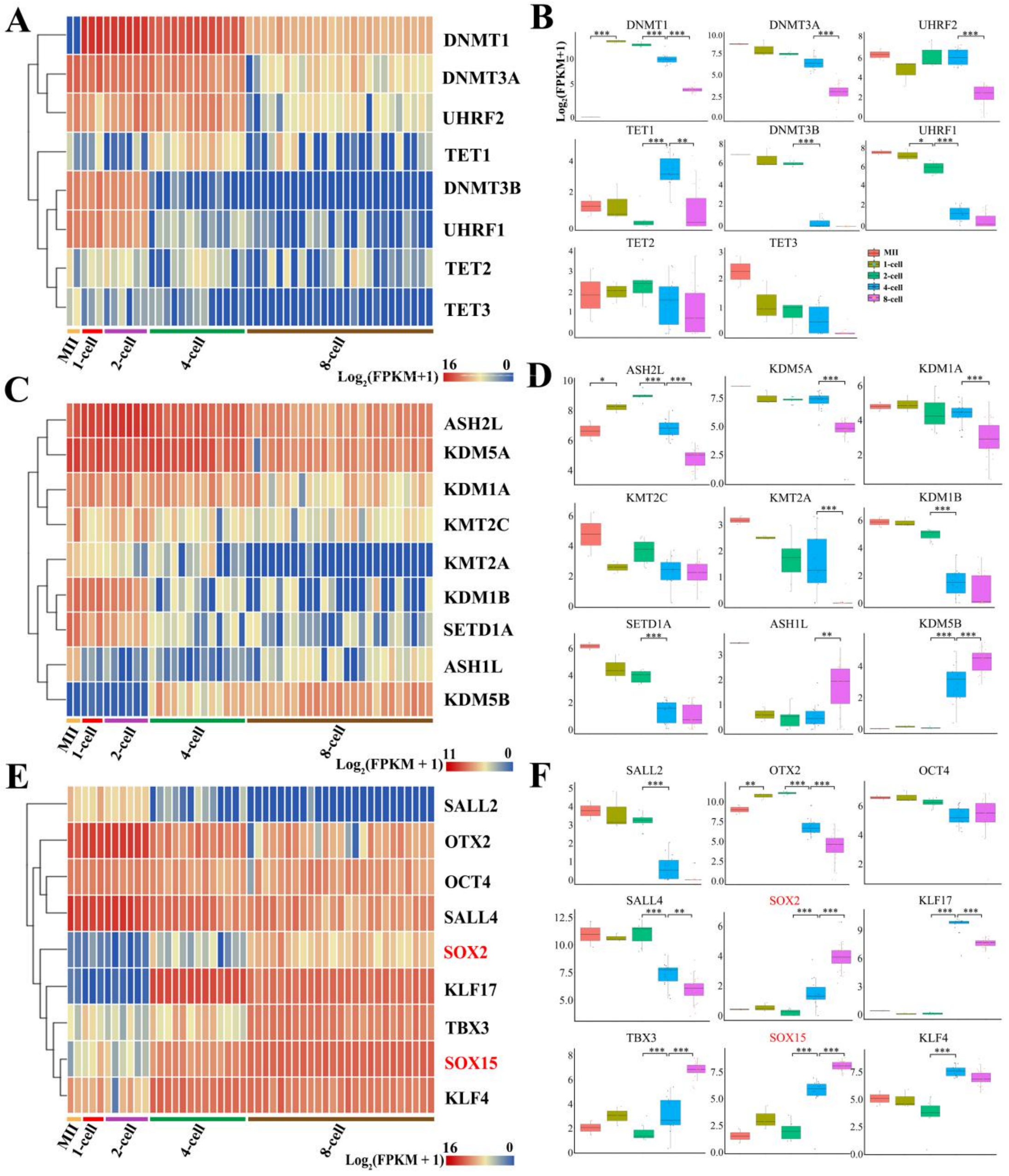

Figure S5. Abundance of mRNAs related to DNA methylation, H3K4 methylation and transcription factors in pig in vivo embryos (extracted from Table S1 in (Kong et al., 2020))**.** Heatmap (A, C, E) and expression dynamics (B, D, F).
